# Osteocalcin—A Versatile Bone-Derived Hormone

**DOI:** 10.3389/fendo.2018.00794

**Published:** 2019-01-10

**Authors:** Sarah C. Moser, Bram C. J. van der Eerden

**Affiliations:** Department of Internal Medicine, Erasmus MC, Rotterdam, Netherlands

**Keywords:** osteocalcin, osteoblasts, bone, energy metabolism, exercise, cognition, fertility, endocrine regulation

## Abstract

Bone has long been regarded as a static organ, simply providing protection and support. However, this mindset has changed radically in recent years and bone is becoming increasingly recognized for its endocrine function of secreting several hormones, thereby controlling various physiological pathways. One of the factors released by the skeleton is osteocalcin. Importantly, osteocalcin is secreted solely by osteoblasts but only has minor effects on bone mineralization and density. Instead, it has been reported to control several physiological processes in an endocrine manner, such as glucose homeostasis and exercise capacity, brain development, cognition, and male fertility. The aim of this review is to provide an overview of the currently known roles of osteocalcin and their underlying mechanisms. At present, one of the major goals in this field is translating basic research into therapeutic applications, therefore ongoing efforts to bring these findings to the clinics will also be discussed.

## Introduction

Bone is well-known for its established role as connective tissue providing not only support and protection for vital organs but also mobility to the body. These functions are known to be exerted by the three major cell types of the skeleton: osteoblasts, osteoclasts, and osteocytes. Osteoblasts, originating from skeletal stem cells, are responsible for synthesis and secretion of type I collagen to synthesize and maintain the extracellular matrix and are able to control osteoclast differentiation. Beside this thoroughly studied role, a new line of research has emerged in recent years, suggesting that osteoblasts secrete factors possessing hormonal function and are thereby able to control other organs. Based on this, numerous studies have been performed, proposing that bone is an essential endocrine organ controlling a panoply of physiological processes, such as energy metabolism, adipogenesis, neuronal development, muscle growth, and male fertility.

One of the key players in bone endocrinology is osteocalcin, or bone γ-carboxyglutamic acid protein, a factor expressed and secreted solely by osteoblasts (1). Following protein synthesis, the mature peptide first undergoes several splicing events and then gets γ-carboxylated at three residues, resulting in a peptide with high affinity toward bone and the extracellular matrix (2). Due to the low pH inside the osteoclast resorption compartments, however, osteocalcin gets decarboxylated again, which reduces its affinity for bone and triggers the release of uncarboxylated osteocalcin into the circulation (3).

Initially, osteocalcin was hypothesized to act in extracellular matrix mineralization and was used as a serum marker of osteoblastic bone formation (4). However, in the late 90s osteocalcin depletion was found to have minor effects on bone density and mineralization in mice (5). Since then, great efforts have been made to determine the actual function of this protein. Up to now, several roles of osteocalcin have been revealed and current reports, which are mainly based on murine and *in vitro* studies, suggest that the uncarboxylated form of osteocalcin controls physiological pathways in an endocrine manner.

**Figure 1:**
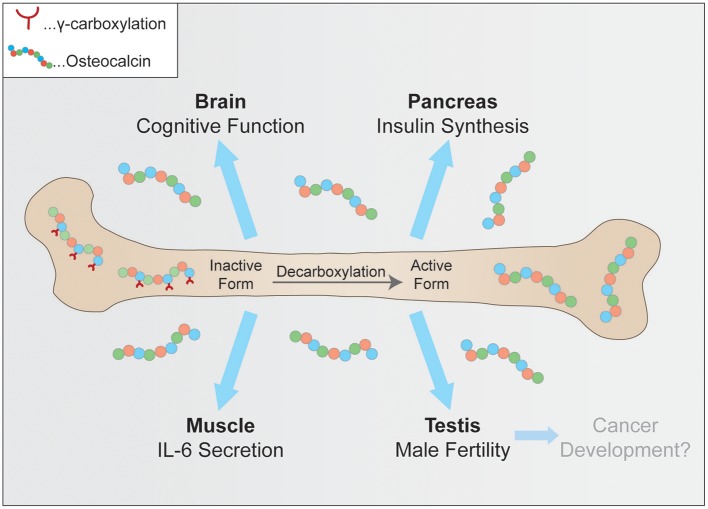
Physiological pathways controlled by osteocalcin. Uncarboxylated osteocalcin is released by osteoclasts and then binds its receptors to influence cognitive function, insulin synthesis in pancreatic β-cells, coinciding with exercise adaptation and testosterone secretion.

The purpose of this review is to summarize the currently known key functions of osteocalcin, which are its role in glucose metabolism and adaptation to exercise, neuronal development, male fertility and a very recent hypothesis linking osteocalcin to tumorigenesis (Figure 1). Furthermore, current efforts to verify these observations in humans and to ultimately translate these findings from basic research into the clinic will be discussed.

### Osteocalcin Regulates Insulin Secretion

In mammals, glucose metabolism is known to be the major source of energy generation. For a long time, it could not be explained why a hormone secreted by the skeleton would be involved in this metabolic pathway. However, looking closer at the ongoing processes in bone, such as bone formation during childhood, the repair of fractures upon injury or the constant remodeling of the skeleton in adults, this clearly indicates that bone demands a significant amount of energy to fulfill its functions. The first evidence of osteocalcin regulating glucose homeostasis was provided from the investigation of osteocalcin-deficient mice in a study by Ducy et al. (5). In contrast to the expected phenotype of bone fragility, mice only showed moderately increased bone mass, but also significantly elevated blood glucose and an increased number of adipocytes together with a higher fat mass compared to their wild-type (WT) littermates (6). Following up on this, the Karsenty group showed that secreted, uncarboxylated osteocalcin induces insulin production in pancreatic islets as well as adiponectin expression in adipocytes, both *in vitro* and *in vivo* (6–8). Further confirmation of this mechanism was provided by a different group, proposing that deletion of the insulin receptor in osteoblasts mimics the observed phenotypes of osteocalcin deletion (9). These findings support the idea of an endocrine bone-pancreas loop where insulin signaling induces osteocalcin expression in osteoblasts, which further stimulates insulin secretion in pancreatic islet cells (9). This hypothesis is further strengthened by studies reporting that insulin sensitivity and glucose tolerance in mice kept on a high fat diet could be restored by daily injections of osteocalcin (10, 11). Whether this feedback loop is also functional in humans is still poorly understood. Until now, osteocalcin has been shown to stimulate human β-cell proliferation *ex vivo* (12) and polymorphisms in the human *Bglap* (osteocalcin) locus have been associated with type 2 diabetes mellitus (T2DM) and obesity (13, 14). Furthermore, several retrospective studies have suggested osteocalcin to negatively correlate with fat mass in several nationalities and age groups (15–19) and to positively correlate with insulin sensitivity and adiponectin levels (20). It is critical to mention though that these studies only investigated subgroups of patients, such as T2DM patients and specific age groups, which may favor the detection of correlations even if relatively weak. Moreover, total levels of osteocalcin are frequently used as a read out in clinical studies, whereas murine studies have clearly shown that it is mainly the uncarboxylated form influencing glucose metabolism. A distinction between these two forms will be critical to detect possible functional relationships. Importantly, confirming the role of osteocalcin during glucose metabolism in humans may have direct therapeutic implications for T2DM patients. All in all, the role of osteocalcin in glucose metabolism is not yet fully supported by human studies due to the above-mentioned pitfalls.

### A Role for Osteocalcin in Brain Development and Cognitive Function

Apart from the well-studied role of osteocalcin in energy homeostasis, it was also found to regulate proper brain development and function. Interestingly, osteocalcin-deficient mice were found to be more passive and to suffer from increased anxiety and decreased memory compared to their WT littermates. Also anatomically, the brain, especially the hippocampus, was shown to be smaller and less developed. (21). Osteocalcin was described to regulate the synthesis of neurotransmitters in the brain, such as dopamine, serotonin, or norepinephrine. Importantly, these altered expression levels and behavioral phenotypes could be rescued by injecting mice with uncarboxylated, active osteocalcin but the developmental defects in the brain remained (21). Therefore, osteocalcin-deficient (*Ocn*^−/−^) mice were crossed and osteocalcin was supplemented to the mothers, which alleviated the poor neuronal development of the hippocampus. This experiment suggests that osteocalcin is required during embryonic development for proper neuronal development and is not synthesized by the embryo itself but provided by the mother. The fact that osteocalcin is able to rescue memory loss and defective neuronal development also sparked interest in the medical field. Considering the fact that Western society is aging disproportionately and increasing numbers of people are suffering from cognitive decline, osteocalcin may be a promising, novel therapeutic agent to alleviate these symptoms. Interestingly, murine studies are already pointing toward that direction because all symptoms of *Ocn*^−/−^ mice can be rescued by exogenously providing uncarboxylated osteocalcin (21). Additionally, a recent report proposed that plasma injections from WT mice, as well as mini osmotic pumps delivering osteocalcin over a longer period of time, could rescue the cognitive defects and anxiety-related features in *Ocn*^−/−^ mice (22). Further mechanistic investigations suggest that the target receptor of osteocalcin in the brain is the G-protein coupled receptor *Gpr158*. However, this finding should be handled with caution, as *Gpr158* is not expressed in all the brain regions where osteocalcin is active, indicating that other receptors and mechanisms are contributing to osteocalcin signaling in the mouse brain. In order to validate this novel role for osteocalcin in humans, several follow-up studies have been performed to find correlations between osteocalcin and cognitive performance in humans. According to current literature, though, a possible correlation in humans is still under debate. On the one hand, reports have been published stating a positive correlation between osteocalcin levels and cognitive performance in aged women (23) and obese patients (24), but on the other hand a recent study in aged individuals with memory concerns (25) could not observe any association with either uncarboxylated or total osteocalcin levels. Considering that the initial finding is very recent, the coming years will bring more studies looking for correlations between osteocalcin levels and cognitive function. Considering this severe effect of osteocalcin on cognitive function, the question arises why a bone-derived hormone may control the brain. Literature indicates that the brain controls bone mass acquisition and that patients suffering from neuropsychiatric disorders often suffer from osteoporosis or low bone mass (26, 27). Furthermore, mice lacking osteocalcin have also been reported to suffer from decreased dopamine and serotonin levels. This suggests that as in glucose metabolism, osteocalcin may function as a feedback signal to the brain to stimulate bone mass acquisition. However, further investigations are required to better investigate this hypothesis. Moreover, larger studies looking at participants differing in age, sex, health status, and ethnic heritage are needed to draw a definite conclusion and will reveal whether osteocalcin is a promising target for aging-related diseases.

### Accurate Control of Osteocalcin Levels Is Essential for Male Fertility and Cancer Prevention

Since sex hormones are known to be important regulators of bone strength during several stages of life, such as adolescence or menopause, it seems likely that osteocalcin may also function in a feedback loop in this metabolic process. Active osteocalcin signaling might indicate bone strength and health, which together with many other fitness markers in the body may contribute to fertility. From an evolutionary perspective this would then favor healthy and strong individuals in order to generate offspring. Indeed, *Ocn*^−/−^ mice were found to have significantly smaller litter sizes when compared to WT animals. By performing co-culture experiments using ovary and testis explants incubated with supernatants of mouse osteoblasts, osteoblasts were found to strongly induce testosterone secretion but not estradiol or progesterone production. To further confirm that it is indeed osteocalcin mediating this effect, testosterone-secreting Leydig cells were incubated with supernatants of WT or *Ocn*^−/−^ osteoblast cultures. Interestingly, the supernatants lacking osteocalcin were no longer able to induce testosterone secretion. Furthermore, supplementing Leydig cells with uncarboxylated osteocalcin increased testosterone secretion in a dose-dependent manner. Switching to an *in vivo* model, Oury and colleagues reported that *Ocn*^−/−^ mice suffer from low testosterone levels and reduced weight of the reproductive organs and oligospermia, which could be rescued by supplementing the mice with exogenous, uncarboxylated osteocalcin (7). In the process of deciphering the molecular mechanism, this study and others identified the receptor required for osteocalcin-mediated testosterone secretion: the G-protein coupled receptor GPRC6A, which was also found to be critical for osteocalcin controlling insulin secretion (7, 8, 28). For further studies, *Gprc6a*^−/−^ mice were generated and were found to display the same symptoms as *Ocn*^−/−^ mice, including poor breeding and altered insulin sensitivity. However, no behavioral abnormalities were observed, which corroborates previous reports postulating that osteocalcin signaling in the brain is mediated by GPR158 (22). To further investigate this, two patients carrying a missense mutation in the *Gprc6a* locus were characterized. Confirming the observations made in mice, these individuals also suffered from testicular failure and showed increased glucose tolerance (29). Importantly, the reason why this effect of osteocalcin on fertility is only observed in males remains elusive. Therefore, it is essential that these findings are reproduced in independently generated mouse models and by other groups to rule out the cause for these sex differences. A query in the current literature underscores the importance of an accurate regulation of osteocalcin and *Gprc6a*. Low levels of these two factors result in testicular insufficiency but high serum levels of osteocalcin (30–32) as well as overexpression of *Gprc6a* (33) have been linked to the development of prostate cancer. Furthermore, knocking out *Gprc6a* using CRISPR/Cas9 technology abolished osteocalcin-mediated activation of ERK, AKT, mTOR signaling and cell proliferation in human prostate cancer xenograft models (34). This further emphasizes the importance of osteocalcin regulation and the fact that targeting GPCR6A may provide a novel strategy to inhibit cancer cell migration. Another role of osteocalcin in lung cancer development was recently shown by Engblom et al. They postulate that osteocalcin expressing cells become activated by factors secreted from lung adenocarcinoma, which recruits neutrophils to the site of the tumor and promotes tumor growth. Interestingly, deletion of cells expressing osteocalcin triggered significant tumor suppression (35). However, the authors do not show a direct effect of osteocalcin on tumor progression, so whether it is osteocalcin itself promoting activation of neutrophils or whether another factor expressed by these cells mediates this process, remains elusive. Nevertheless, these studies provide an interesting basis for further investigations concerning the role of osteocalcin in tumorigenesis and a possibility to use osteocalcin as a biomarker for cancer development.

### Osteocalcin Is Required for Optimal Adaptation to Exercise

Since osteocalcin has been reported to regulate glucose metabolism, which provides energy to muscles during exercise, it may be involved in the communication between these two tissues. Initially, osteocalcin levels were found to increase in mice and humans during exercise (36). Furthermore, the Karsenty group demonstrated that osteocalcin levels decline during aging, coinciding with a diminished exercise capacity and a decrease in muscle mass. In order to prove that these phenotypes are caused by osteocalcin, mice were injected with uncarboxylated osteocalcin, which could rescue the exercise capacity of these mice and partially reverse the age-dependent decrease in muscle weight (37). Next, a mouse model specifically lacking the osteocalcin receptor *Gprc6a* in muscle cells was generated (*Gprc6a*${{}_{Mck}}^{-/-}$) to assess whether these findings are caused by a direct effect of osteocalcin on muscle cells. Injections of osteocalcin could not increase the exercise capacity of these animals, indicating that osteocalcin signaling is specifically required in myofibers for exercise adaptation. Furthermore, Mera and colleagues stated that osteocalcin signaling supports glucose and fatty acid utilization during exercise, which is in line with previous reports showing that muscle glucose uptake was increased upon exposure to osteocalcin *in vitro* (38, 39). Transcriptome analysis of *Gprc6a*${{}_{Mck}}^{-/-}$ mice revealed that the myokine Interleukin-6 (IL-6), which is secreted by skeletal muscles into the blood upon exercise (40), was found to be significantly downregulated. Therefore, the authors suggested that osteocalcin binds its receptor GPRC6A in muscle cells and is required for ATP generation during exercise by controlling the uptake of glucose and fatty acids. Moreover, a feedforward loop between muscle and bone may exist where IL-6 is secreted from the muscle in order to further stimulate glucose and fatty acid production and the secretion of undercarboxylated osteocalcin. However, further research is needed to elucidate this mechanism of regulation. From a translational point of view, multiple clinical studies already suggest that osteocalcin levels are elevated upon exercise in different gender and age groups (41–44). Furthermore, uncarboxylated osteocalcin levels in women above 70 years have been reported to positively correlate with muscle strength (45). This suggests that osteocalcin and its receptor may be a promising target to combat the age-related decline of muscle strength or to alleviate muscle diseases. Considering that this role of osteocalcin has been reported only very recently, further mechanistic studies are needed to prove that this feedforward loop is also present in humans, and more clinical studies are required to draw definite conclusions about possible therapeutic applications in bone-muscle communication.

## Conclusion

The literature presented above provides evidence that osteocalcin is not only involved in bone remodeling but plays a critical function in multiple physiological processes. Importantly, these findings should be handled with care because most initial studies were performed by one laboratory and are still waiting to be reproduced by other research groups. The fact that osteocalcin is solely regulating male fertility and does not affect female reproduction seems especially counterintuitive and requires further research. Moreover, many discoveries are still lacking mechanistic insight and thorough validation in humans. Considering the highly standardized conditions of murine studies and the high genetic similarity of animals, these findings may not accurately reflect the human situation. Furthermore, most clinical studies investigate groups predefined by age, ethnicity, or gender, which may favor the detection of a relationship. It is also worth mentioning that correlation does not imply a direct function for osteocalcin in humans, therefore investigating larger patient cohorts as well as thoroughly executed genetic studies are needed to gain more mechanistic insights. Given the rapidly advancing field of omics technologies, such as genome-wide screening platforms in combination with powerful next-generation sequencing technology and the ability to study cellular processes at single-cell level, it is feasible that new studies will shed light on the role(s) of human osteocalcin soon. Furthermore, these techniques may aid in discovering previously unknown roles of osteocalcin. Considering the strong regulatory effects of osteocalcin on various organs of our body, it is highly likely that other organs or pathways will be affected by osteocalcin signaling as well. Even when assuming that human data correlate well with the observations made in the initial studies, it may still be a laborious journey until clinical trials are initiated, using osteocalcin to treat metabolic diseases such as T2DM or obesity.

## Author Contributions

SM performed the literature research, wrote the manuscript, and designed the figure. BvdE revised the manuscript.

### Conflict of Interest Statement

The authors declare that the research was conducted in the absence of any commercial or financial relationships that could be construed as a potential conflict of interest.
